# Single-Walled Carbon Nanotube Supported PtNi Nanoparticles (PtNi@SWCNT) Catalyzed Oxidation of Benzyl Alcohols to the Benzaldehyde Derivatives in Oxygen Atmosphere

**DOI:** 10.1038/s41598-020-66492-x

**Published:** 2020-06-15

**Authors:** Haydar Göksu, Kemal Cellat, Fatih Şen

**Affiliations:** 1 Kaynasli Vocational College, Düzce University, Düzce, 81900 Turkey; 2 Sen Research Group, Department of Biochemistry, Dumlupınar University, 43100 Kütahya, Turkey

**Keywords:** Biocatalysis, Synthetic chemistry methodology

## Abstract

This study reports a developed process which is a general and facile method for the oxidation of benzyl alcohol (BnOH) compounds to the benzaldehyde (BA) derivatives, under mild conditions. The oxidation of BnOH species catalyzed by PtNi@SWCNT in toluene (3 ml) at 80 °C under a continuous stream of O_2_. Single wall carbon nanotube supported PtNi (PtNi@SWCNT) nanoparticles were synthesized using a single-step modified reduction process. The characterization of PtNi@SWCNT nanocatalyst was performed by transmission electron microscope (TEM), X-ray diffraction (XRD), X-ray Photoelectron Spectroscopy (XPS), and elemental analysis by ICP-OES. A variety of BnOH compounds were oxidized by the PtNi@SWCNT catalyst and all the expected oxidation products were obtained in high efficiency in 2 hours of reaction time. TLC was used to monitoring the reaction progress, and the products were identified by ^1^H/^13^C-NMR analysis.

## Introduction

The bimetallic nanoparticles have been recently extensively studied in many applications such as fuel cells, electrochemical sensors, biosensors, solar cells, drug materials, hydrogen storage, etc.^1–6^. As a catalyst, bimetallic nanoparticles have many unique properties such as increased efficiency, activity, selectivity, durability, reusability, stability, etc. Those properties are explained by the synergistic effect of two different metal atoms. The catalytic activity is related to the size and shape of the nanoparticles, the interactions between the metal atoms, and the support material. Bimetallic catalysts, basically simplify a complex synthesis and are used to synthesize valuable molecules with higher efficiency, has been used for alcohol oxidation, including fuel cells in recent years^7,8^. On the other hand, the oxidation of primary alcohols is one of the most important tools in organic chemistry for the synthesis of carboxylic acids, aldehydes, and ketones. Even numerous oxidation methods have been developed such as chromium and manganese oxides, hypervalent iodine reagents, pyridine·SO_3_, and NaOCl/TEMPO (TEMPO = 2,2,6,6-tetramethyl-1-piperidinyloxyl); the oxidation by O_2_ gas is still common method in research laboratories and industry. Numerous studies for the oxidation of primary alcohols can be found in the literature. Most of these studies are based on the conversion of primary alcohols to carboxylic acids and esters. However, keeping the aldehydes of primary alcohols is quite difficult and requires special systems. Especially in the presence of water, the aldehyde phase of the alcohols in the oxidation stage is not achieved. Since the aldehydes obtained by one stage oxidation of primary alcohols are highly reactive molecules and continue to oxidize, forming carboxylic acids or esters. Therefore, aldehyde compounds formed in the reaction medium should be either rapidly removed from the reaction medium or reactions should be maintained in a non-aqueous medium^9–13^.

In the oxidation reactions, organic (nitrogen-containing) and inorganic (K_2_CO_3_, Na_2_CO_3_, NaCH_3_COO, KCH_3_COO, CoCl_2_) bases are generally utilized^14,15^. However, these oxidation reactions can be conducted for specific compounds only. As a result of some disadvantages such as slow reaction time, low efficiency, and weak selectivity; their practical applications are limited. Thus, a novel eco-friendly oxidation process, not only allows the oxidation of a variety of BnOH but also exhibits high selectivity and efficiency, is still required.

Pt is one of the main catalysts used in catalytic oxidation reactions in organic chemistry. Platinum is a noble metal, and the price of Pt is quite high. Hence, it is necessary to develop less expensive Pt-based catalysts and improve its overall catalytic performance. For this aim, bimetallic and trimetallic catalysts can be used, and this approach not only provides a more cost-effective solution but also gives better performance compared to monometallic catalysts. Moreover, a variety of carbon supports including carbon microspheres^16^, carbon nanotubes^17^, carbon nanofibres^18^, carbon blacks^19^, activated carbon (AC)^20^, graphene oxide^21,22^ have been widely used in heterogeneous catalysis. Recently, graphene oxide supported γ-MnO_2_ nanocomposites^23^, metal oxide supported gold nanoparticles^15,24^, and hydroxyapatite-supported palladium nanoclusters^25^ have been used as heterogeneous catalysts for the oxidation of BnOH compounds to benzaldehyde derivatives. The BnOH compounds were easily converted to aldehyde derivatives with satisfying yields. The alcohol groups were selectively oxidized to BA derivatives without conversion to carboxylic acids and esters. Due to some disadvantages of commonly used support materials, there is still a need for performing new researches to develop novel nanostructured carbon-based catalysts with good chemical and physical properties. Single-walled carbon nanotubes (SWCNT) as a support material for metal catalysts are favorable due to their high mechanical strength, stability, high surface to volume ratio, and selectivity^26^.

This work reports a general and facile method for the oxidation of BnOH species to the aldehyde derivatives under mild conditions. The oxidation of primary alcohols catalyzed by PtNi@SWCNT nanoparticles in 3 ml of toluene 80 °C. PtNi@SWCNT nanoparticles were synthesized using a single-step modified reduction method. A variety of BnOH mixtures were tested by PtNi@SWCNT nanoparticles as a catalyst. All the expected oxidation products were achieved by the very high selectivity (up to 100%) in short reaction times of about 2 hours.

## Experimental

### Materials

The standard airless procedure was used for the synthesis. All the reagents were used without any purification. PtCl_4_ (99%) was purchased from Alfa-Aesar. Ni(CO_2_CH_3_)_2_ 4H_2_O (98%), ferrocene (98%), thiophene (≥99%), dimethylamine borane (DMAB, 97%) were obtained from Sigma-Aldrich, and ethanol (99.9%) were obtained from Merck. All the BnOH compounds were obtained from Sigma-Aldrich.

### Characterization methods

The characterization of PtNi@SWCNT nanoparticles was achieved using, XRD, XPS, and TEM analyses. The X-ray diffraction patterns (*θ*–2*θ* scans) were taken on a Panalytical Empyrean diffractometer with Ultima+ theta–theta high-resolution goniometer system using Cu Kα (*λ* = 1.54056 Å) as the X-ray source. Scans were recorded at 40 kV and 40 mA, in step scan mode between 2θ range of 20 and 90° (scan rate 0.02° 2θs^−1^).

X-ray Photoelectron Spectroscopy (XPS) measurements were carried out in a Specs spectrometer instrument. The X-ray source was Mg Kα (1253.6 eV, 10 mA) radiation. Cu double-sided tape (3 M Inc.) was used for the sample preparation. The reference point was C 1 s line at 284.6 eV. TEM images of PtNi@SWCNT nanoparticles have been obtained by a JEOL 200 kV TEM instrument. ^1^H and ^13^C NMR spectra were recorded on a Jeol ECS 400 MHz spectrometer. All the individual NMR spectra were analyzed using JEOL Delta NMR control and process software.

Inductively coupled plasma optical emission spectrometry (ICP-OES, Perkin Elmer Avio 500 coupled with Ultrasonic Nebulizer) analyses were performed to concentration determination of Pt and Ni after consecutive using of PtNi@SWCNT nanoparticles. The operating parameters of the ICP-OES were as follows: nebulizer flow of 10 mL min^−1^; an auxiliary gas flow of 0.2 L min^−1^; sample introduction of 1 mL min^−1^; in radial mode.

Thin-layer chromatography (TLC) was performed on 20 × 20 cm plates pre-coated with silica gel, and the mobile phase was ethyl acetate/hexane (v:v = 1:5).

Organic compounds were determined by gas chromatography-mass spectrometry (GC/MS, Agilent) equipped with a mass selective detector and HP-5MS column (30 m, 250 μm, 0.25 μm). The operation conditions of GC/MS: an ion source, the mass spectrum of the cations produced by 70 eV electron impact ionization; 280 °C of transfer line temperature; carrier gas was He (purity ≥99.99%) with a flow rate of 1 mL/min. Splitless injection mode with 1 µL sample solution was used. The oven temperature program was as follows: hold initial temperature at 100 °C for 0 min, 10 °C/min to 250 °C 31 min, 4 °C/min to 280 °C, hold at 280 °C for 31 min. The compounds were identified according to the mass spectra and their retention time. Results were compared to internal reference library data.

### Synthesis of SWCNT

For the SWCNT synthesis, ferrocene was used as a catalyst precursor, thiophene was growth promoter, and ethanol was carbon source. Ferrocene (0.3 wt%) and thiophene (molar ratio of S/Fe = 0.2) were dissolved in ethanol (99.9%) and placed in an ultrasonic bath for 2 minutes to obtain a homogenous solution. The synthesis was conducted by injection of the solution using a syringe pump at a feed rate of 4–10 µL/min. The resulting solution was evaporated in a heating line at a constant temperature of 140 °C. It was carried via a mixture of H_2_ and N_2_ gases with a volume of 800 sccm. The SWCNTs particles were collected at the downstream of the reactor and filtered using a membrane filter^27^.

### Synthesis of PtNi@SWCNT nanoparticles

A facile, single-step modified reduction procedure was employed for the synthesis of PtNi@SWNT nanoparticles. Initially, 60 mg of as-synthesized SWCNT, 30 mg of Ni(CO_2_CH_3_)_2_ 4H_2_O, and 30 mg of PtCl_4_ were dissolved in absolute ethanol. The mixture was kept in a sonicator for 30 min to achieve a homogeneous solution. The mixture was poured into a Schlenk tube and mixed under N_2_ gas flow for 1 h, it was mixed for 45 min after the addition of DMAB (148 mg). Subsequent to the mixing procedure, it was refluxed, and the resulting nanoparticles were isolated by centrifugation. The obtained black PtNi@SWCNT nanoparticles were washed with purified water, and dried in a vacuum oven at a temperature of 150 °C, and used as a catalyst for the redox reactions in this study.

### PtNi@SWCNT catalyzed reduction of BA compounds

NaBH_4_ (3.0 mmol) was added to a mixture of BA (1 mmol) and PtNi@SWCNT nanoparticles (2 mg) in 3 mL of water/methanol (v/v = 1/2) and the reaction mixture was stirred at room temperature. The reaction progress was checked by TLC. The products were identified by ^1^H/^13^C-NMR analysis. The yields stated in the article are not isolation yields.

### PtNi@SWCNT catalyzed oxidation of BnOH compounds

To a mixture of BnOH (1 mmol) and PtNi@SWCNT nanoparticles (2 mg) in toluene (3 ml) was added KOH (1.5 mmol) and the reaction mixture was stirred at 80 °C under a continuous stream of O_2_ for the required time. The reaction progress was observed by TLC. The products were identified by ^1^H/^13^C-NMR analysis. The yields stated in the article are not isolation yields.

## Results and Discussion

**Figure 1:**
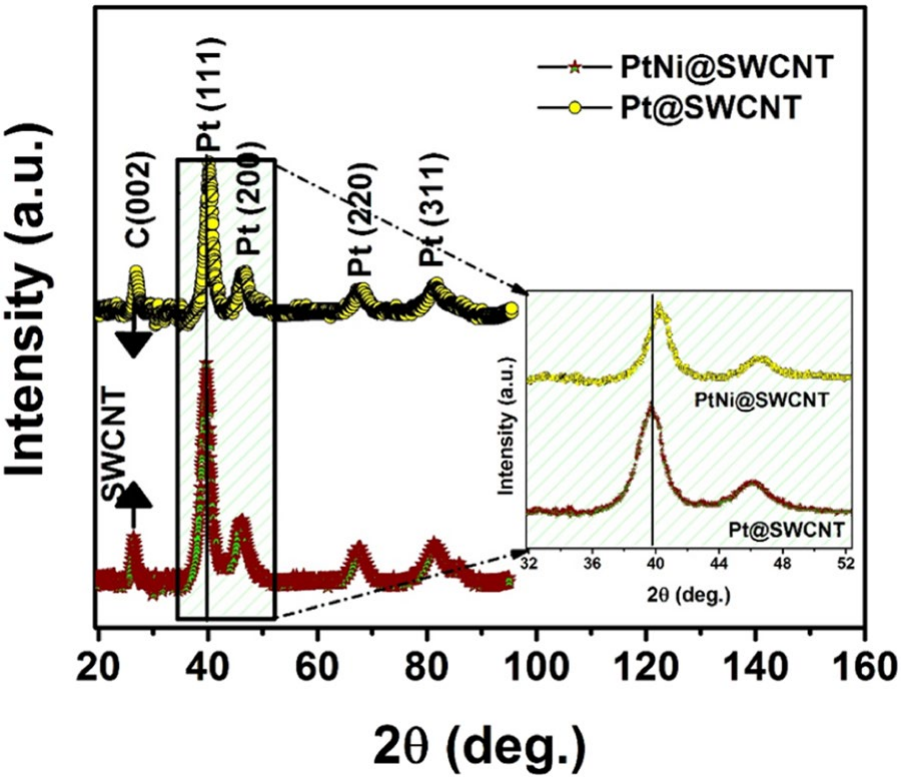
X-ray diffractograms (XRD) of Pt@SWCNT and PtNi@SWCNT nanoparticles.

The crystalline structure of the PtNi@SWCNT was investigated by XRD analysis (Fig. 1). The XRD data exhibited clear diffraction peaks of PtNi@SWCNT. The diffraction peak observed at around 25.1° corresponds with the carbon (0 0 2) reflection of the graphitic planes of the SWCNT (JCPDS card no. 75-1621). The peaks appearing at diffraction angles of 39.8°, 46.2°, 67.7°, 81.5°, and 83.8° (JCPDS-ICDD, Card No. 04-802) are corresponding to the face-centered cubic crystal (fcc) planes of (1 1 1), (2 0 0), (2 2 0), (3 1 1), (222) respectively. There are no noticeable characteristic peaks for Ni and its oxides; however, their existence cannot be discarded due to the fact that they may be present as very small crystallites, and alloy formation between Pt and Ni. In addition, a right shift to higher 2θ values was detected on PtNi@SWCNT peaks, compared to Pt@SWCNT, in agreement with Vegard’s law^28^, and this is a result of the substitution of smaller Ni atoms.

**Figure 2:**
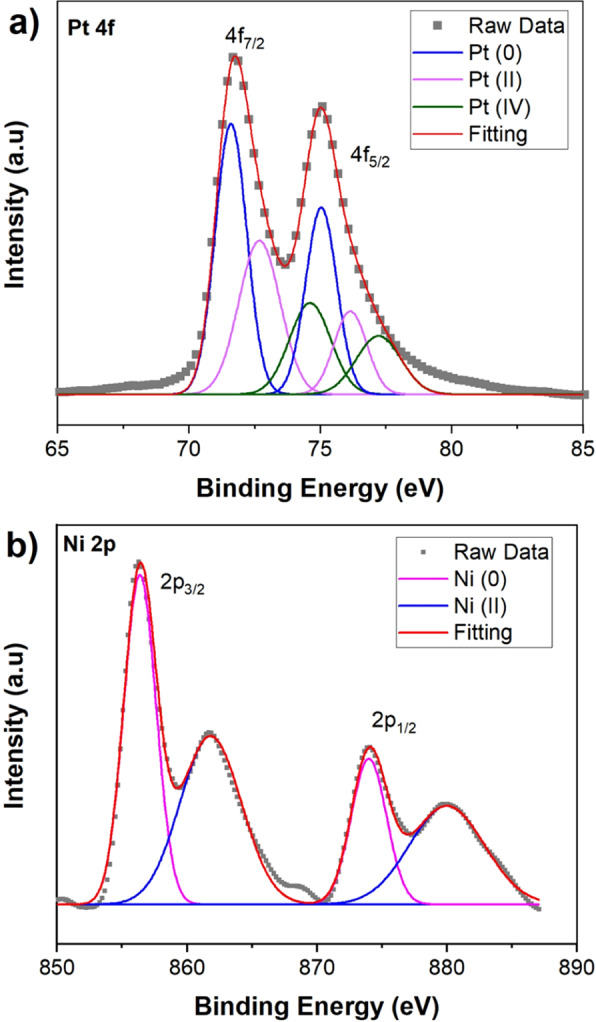
XPS of PtNi nanoparticles. (**a**) 4f core level spectrum in Pt, (**b**) 2p core-level spectrum in Ni.

In electrocatalyst studies, X-ray photoelectron spectroscopy (XPS) is an analytical technique for the determination of changes in the electronic structure based on shifts of the binding energies of core-level electrons. The XPS spectrum of the PtNi@SWCNT was shown in Fig. 2. The XPS peaks were fitted using the Gaussian–Lorentzian method, and the Shirley-shaped background correction procedure was applied. The peaks of Pt 4f_7/2_ and Pt 4f_5/2_ spectrum appearing at a binding energy of 71.5 eV and 75.0 eV correspond to metallic Pt°. (Fig. 2a). The peaks for Pt^2+^ species, such as PtO and Pt(OH)_2_, were observed at 72.7 eV and 76.2 eV, and Pt^4+^ species were detected at 74.6 eV and 77.2 eV. According to relative intensities of the peaks, Pt is predominately present in the metallic state (Pt°). The peaks for Ni 2p_3/2_ and Ni 2p_1/2_ with the binding energies of 856.3 eV and 874.0 eV are characteristic of metallic Ni° (Fig. 2b). The other peaks on Ni 2p spectrum at 861.8, 880.1 eV correspond to Ni^2+^. The characteristic peaks observed in XPS spectra prove the existence of Pt and Ni in the samples. With reference to pure Pt, the Pt 4 f XPS spectrum of PtNi nanospheres experienced peak shifts negatively, which indicates an electronic stage change of Pt when it was alloyed with Ni.

**Figure 3:**
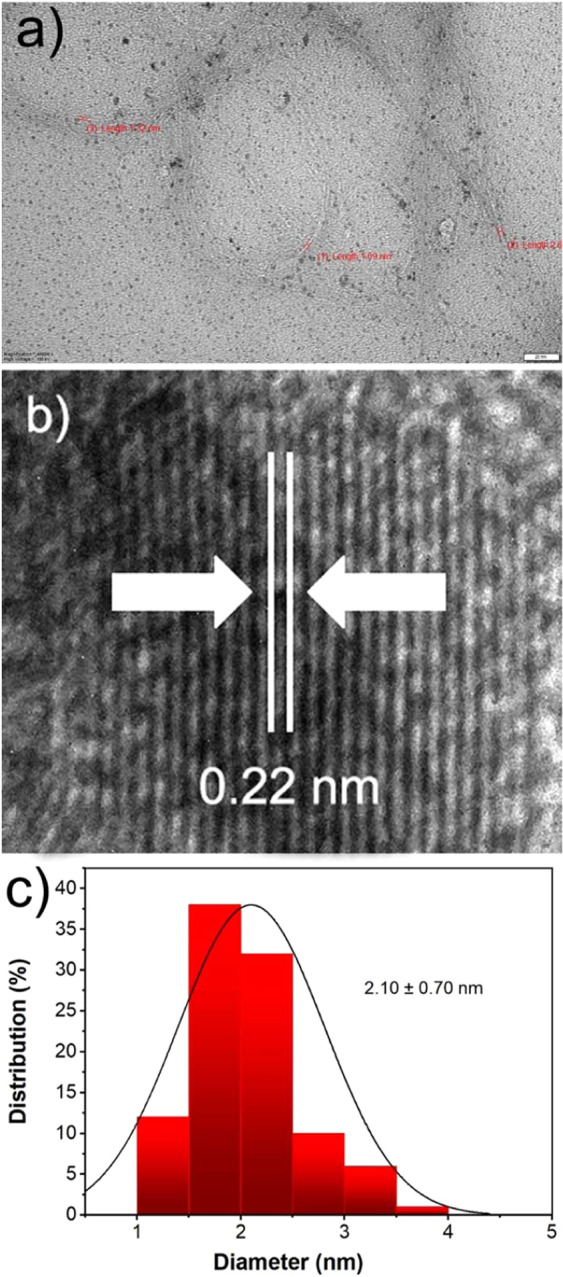
(**a**)TEM and (**b**) HRTEM image, (**c**) particle size disturbution of PtNi@SWCNT.

TEM analysis was carried out to determine the morphological structure and particle size of PtNi@SWCNT (Fig. 3). The particles were largely spherical, homogeneously distributed on the SWCNT support, and no agglomeration was observed. As shown in Fig. 3(a), the diameter of SWCNTs was found to be 1.3‒2.8 nm. The representative atomic lattice fringes of PtNi@SWCNT was measured as 0.22 nm (Fig. 3(b)), which is quite close to the nominal Pt(111) spacing of 0.23 nm. Furthermore, particle size histogram obtained from counting approximately 300 particles was given in Fig. 3(c) and the mean particle size was calculated as 2.10 ± 0.70 nm. The particle size analysis is compatible with the previous studies in literature^29^.

The selective oxidation performance of PtNi@SWCNT nanocatalyst on BnOH to BA was examined. The performance tests were conducted in the 5 wt% of oxidant catalyst loading; in the presence and absence of a base, K_2_CO_3_, NaHCO_3_, KOH; at room temperature, and 80 °C (Table 1). Despite of the fact that the reaction time was 5 hours, no products were detected without a base. After 4 hours of reaction time, when 1 mmol of K_2_CO_3_ and NaHCO_3_ was used, the yield of BA was 12% and 10%, respectively. Adding 1 mmol of KOH and 4 mg of catalyst into reaction medium significantly increased the conversion yield of the reaction (Table 1, entry 4). 2 mg of catalyst was used, the BA was obtained by high conversion yields within 2 hours using 1.5 mmol of the base (Table 1, entry 7). When there was no catalyst in the reaction medium at room temperature, BA formation was not observed (Table 1, entry 8, 9).

**Table 1:** Optimization studies^a^.


**Entry**	**Catalyst amount (mg)**	**Base**	**Temperature (°C)**	**Time** **(h)**	**Yield**^***b***^ **(%)**
1	4	—	80	5.0	Trace
2	4	K_2_CO_3_ (1 mmol)	80	4.0	8.8 ± 2.9
3	4	NaHCO_3_ (1 mmol)	80	4.0	5.4 ± 4.6
4	4	KOH (1 mmol)	80	4.0	80 ± 2.0
5	2	KOH (1 mmol)	80	4.0	78 ± 2.0
6	2	KOH (1.5 mmol)	80	4.0	>93 ± 1.0
**7**	**2**	**KOH (1.5 mmol)**	**80**	**2.0**	**>99** ± 1.0
8	2	KOH (1.5 mmol)	rt	2.0	Trace
9	—	KOH (1.5 mmol)	80	2.0	Trace

^a^Reaction Conditions: PtNi@SWCNT (5% wt of PtNi), 1.0 mmol substrate, 3.0 mL of toluene, a continuous stream of O_2,_ (N = 3).

^b^GC yield.

The presence of molecular oxygen is important in terms of the continuity and efficiency of oxidation reactions. In this study, the absence of molecular oxygen causes a significant decrease in reaction efficiency. Because the catalyst is regaining its activity with molecular oxygen. It is not sufficient to maintain the reaction in air environment when BA from BnOH is present in the medium. In particular, there is a need to provide a continuous flow of oxygen gas to increase the concentration of molecular oxygen in the reaction medium. After optimization experiments, the BA synthesis procedure was carried out from BnOH. It was then determined which BnOH derivatives to work on. The selected BnOH derivatives were molecules that contained electron donor and electron acceptor groups such as 4-hydroxy benzyl alcohol and 4-nitro benzyl alcohol, respectively, as well as steric effects such as anthracene. The results of PtNi@SWCNT nanoparticles catalyzed NaBH_4_ dehydrogenation and hydrogenation reactions over a variety of BA derivatives were summarized in Table 2. In the BA compounds test series, all the compounds reduced to the corresponding primary alcohols with excellent efficiencies in a short time at room temperature. These experimental results suggest that the PtNi@SWCNT nanoparticles are sensitive to the reduction of aldehyde functional groups. In the first stage, some BA derivatives were reduced to the BnOH compounds by transfer hydrogenation, and PtNi@SWCNT nanoparticles were used in this procedure for the first time in the literature. Finally, 1.0 mmol of BA derivatives, 2.0 mg of PtNi@SWCNT, and 3.0 mmol of NaBH_4_ exhibited satisfactory performance on the reduction of BA derivatives at room temperature with high conversion yields in water/methanol mixture (1:2, 3 mL) (Table 2, entry 1–6).

**Table 2:** The reduction of some of the aromatic nitro compounds using PtNi@SWCNT as a catalyst.


**Entry**	**Substrate**	**Product**	**Conversion (%)**	**Selectivity (%)**
1			>99 ± 1.3	98 ± 1.4
2			>99 ± 1.0	99 ± 1.2
3			>96 ± 2.9	97 ± 2.6
4			>97 ± 1.9	98 ± 2.1
5			>98 ± 1.3	99 ± 1.1
6			>97 ± 2.9	96 ± 3.1

Reaction Conditions**:** 2.0 mg PtNi@SWCNT(5% wt of PtNi), 1.0 mmol substrate, 3.0 mmol NaBH_4_, 3.0 mL H_2_O:MeOH (1:2), room temperature, 10 minute (N = 3).

**Figure 4:**
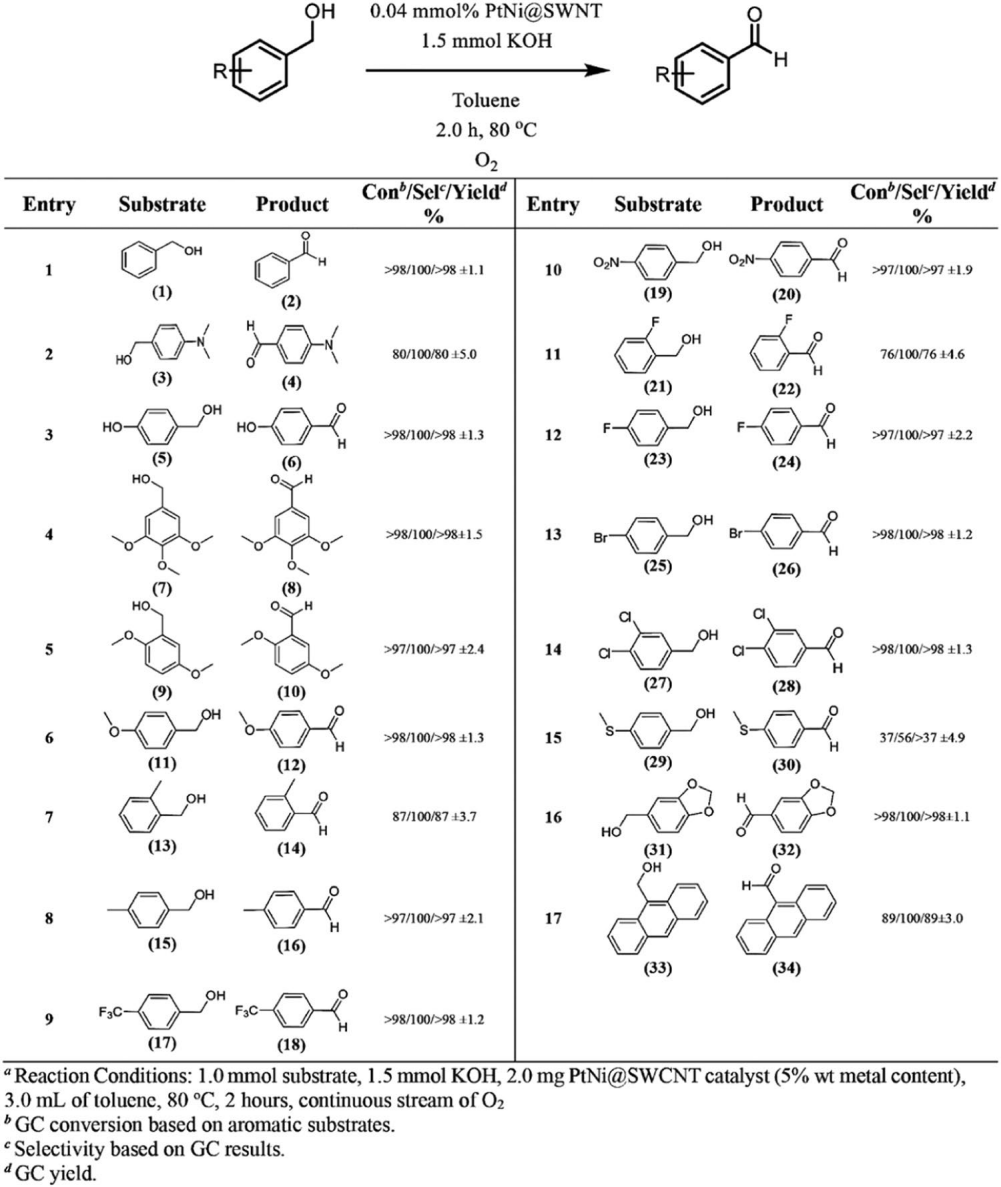
The catalytic performance of PtNi@SWCNT on oxidation different BnOH compounds^a^.

Figure 4 indicates the catalytic performance of PtNi@SWCNT nanoparticles on BnOH oxidation. Herein, individual BA derivatives were successfully achieved with high efficiencies by oxidation of various BnOH compounds in 2 hours at 80 °C. For example, the oxidation of BnOH (1) into BA (2) was obtained with 99% efficiency. (3) (Fig. 4, entry 1). Conversation of 4-(dimethylamino) benzyl alcohol to 4-(dimethylamino)benzaldehyde (4) was achieved with 85% efficiency (Fig. 4, entry 2). The high conversion yields were also obtained in BnOH derivatives which have electron-donor groups such as methyl (-CH_3_), methoxy (-OCH_3_), and hydroxyl (-OH). The product efficiency was increased by bonding activity on the surface of the catalyst (Fig. 4, entries 3–6, 8). However, the steric effect in the reaction center of the methyl group at the 2 positions of the o-tolylmethanol (13) reduces the efficiency of the final product. Therefore, o-tolylmethanol (13) was oxidized into 2-methylbenzaldehyde (14) with a yield of 91% (Fig. 4, entry 7). The high conversion yield was also obtained in the conversation of (4-(trifluoromethyl)phenyl)methanol (17) into 4-(trifluoromethyl)benzaldehyde (18) (Fig. 4, entry 9). benzo[d][1,3]dioxole-5-carbaldehyde (32), 3,4-dichlorobenzaldehyde (28), 4-bromobenzaldehyde (26), 4-fluorobenzaldehyde (24), and 4-nitrobenzaldehyde (20) were synthesized with an efficiency of >99% (Fig. 4, entries 10, 12–14, 16).

**Figure 5:**
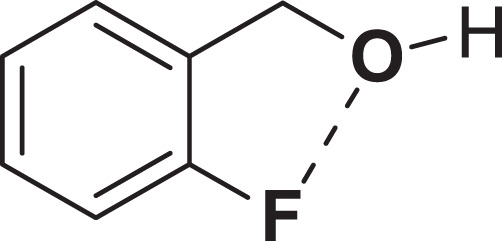
The chemical structure of 2-fluorobenzyl alcohol.

As shown in Fig. 5, a fluorine atom is positioned in the ortho position of 2-fluorobenzyl alcohol (21). Herein, low conversion yields (80%) were obtained due to the coordination of the F atom with the OH group (Fig. 4, entry 11).

Finally, anthracene-9-ylmethanol (33) was converted to anthracene-9-carbaldehyde (34) with 92% efficiency.

**Figure 6:**
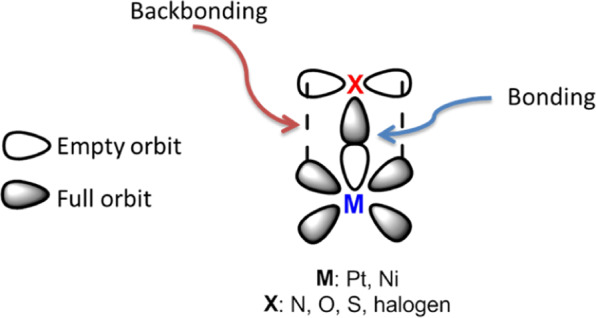
Schematic diagram of back bonding and bonding.

Synergistic bonds are σ and π component bonds. The Sigma component is frequently encountered, and the electron density of the ligand is given to the metal. π component is a part of the electron density of metal that is given to the ligand that is called back bonding (Fig. 6). The metal must have empty trajectories to obtain the electron density of the ligand and must have the appropriate energy and symmetry-filled orbits to restore (back binding) some of the electron density. In addition, the ligand must have electron density and empty π^*^ orbits in the appropriate energy and symmetry to recover some of the electron density of the metal. In the bond establishment, the empty orbitals of metal overlap with the atom or hybrid orbitals containing the electron pair of the ligand. Moreover, the full orbitals of metal match with the π^*^ orbitals of the ligand. The s, p, and d orbitals can be used here.

The binding event, i.e. the σ component, is generally essential between ligand and metal, in catalytic reactions. But, the back bonding is highly related with the existence of ligand’s d orbitals. Accordingly, the molecules are connected to the catalyst surface stronger. If back bonding is not close to the reaction center, reduction in the reaction efficiency is occurred.

Due to the fact that binding event increases the interaction process on the surface of the catalyst and around it of the BnOH derivatives, the increase in the reaction efficiency has occurred. However, due to the limited mobility on the catalyst surface, the reaction efficiency of (4-(methylthio)phenyl)methanol (29) is reduced (Fig. 4, entry 15).

As shown in Fig. 4, the presence of atoms such as halogen, nitrogen, and oxygen in the alkyl groups in the BnOH derivatives increases the conversion yield of the reaction. However, the size of the alkyl groups bound to these heteroatoms ((4-(dimethylamino)phenyl)methanol (3)) or the size of the molecule (anthracene-9-ylmethanol (33)) causes a decrease in the conversion yield of the reaction (Fig. 4, entry 17).

**Figure 7:**
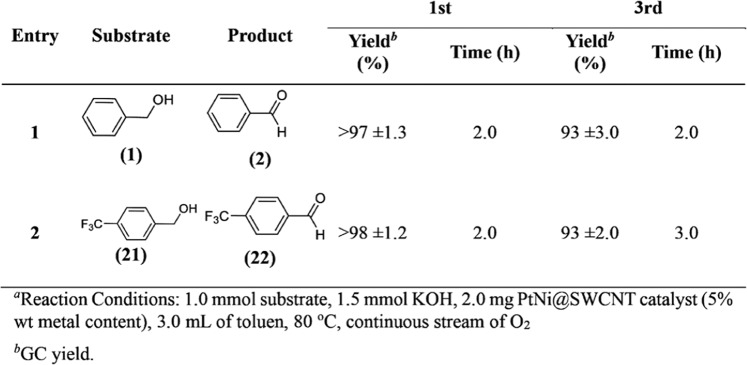
Reusability performance of PtNi@SWCNT nanoparticles^a^.

In addition to high catalytic performance, PtNi@SWCNT is also reusable and stable, after 3^rd^ repeated using ≤95% conversion efficiency was obtained as shown in Fig. 7. Reusability was also proved by the ICP-OES analyses, and no significant loss of Pt and Ni (0.8 and 1.0 ppm leaching to a solution respectively) was observed after three cycles.

**Figure 8:**
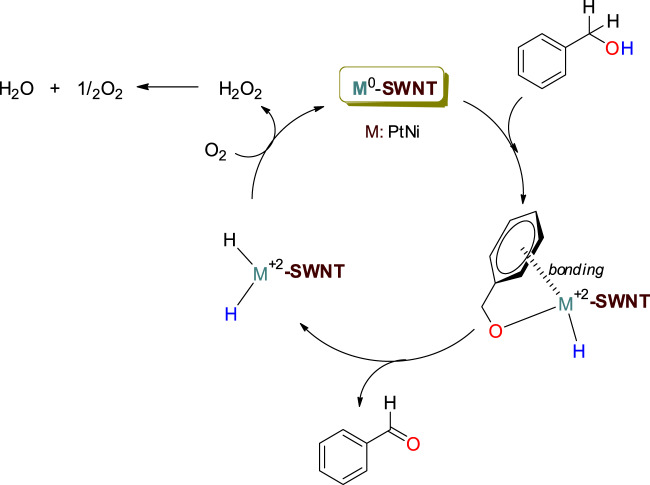
Proposed mechanism for the oxidation reaction.

Figure 8 shows the schematic diagram for the proposed mechanism of oxidation reaction. Initially, the activation of BnOH occurs. Herein, initiating step occurs via coordination of the aromatic ring on the surface of the catalyst and the coupling of the alcohol oxygen to the metal. Then, aldehyde formation is occurred by linking the hydrogen atoms of the hydroxy group and the benzylic position to the catalyst surface. both the coordination and aldehyde formation stages, zero-valent metal is oxidized to +2 state. The oxidized metal ion is re-reduced to zero-valent metal atom in the coexistence of O_2_. As a result of this, catalytic activity is renewed and the oxidation reactions are sustained.

Herein, the coexistence of O_2_, allows the balance between oxidation and reduction to move in the direction of oxidation (Fig. 8). As mentioned in the previous sections, the reaction efficiency decreases in the absence of oxygen. In fact, the final step is inevitable for the hydrogenation reaction, and the resulting BA is converted to BnOH by hydrogenation.

As a matter of fact, our group carried out serious studies on hydrogenation reactions^30–32^ The alcohol derivatives such as glycerol^33–35^, isopropanol^36,37^ and ethanol^38^ have attracted interest as green type alternative hydrogen sources^39–41^.

**Figure 9:**
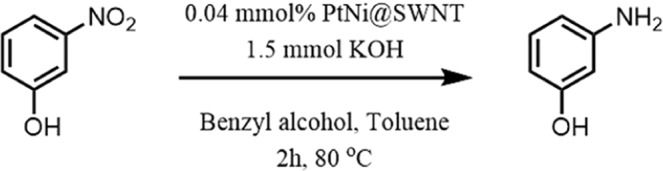
Reduction of the nitro compound in the presence of BnOH as a hydrogen source.

The 3-nitrophenol added to the reaction medium for the test, and it was observed that it converted to 3-aminophenol with a 65% yield in the absence of molecular oxygen (Fig. 9). This result proves the correctness of the idea we suggest.

Eventually, a comparison of the performance of PtNi@SWCNT nanoparticles with previous studies in the literature was given in Table 3 ^14,23,25,42,43^. It was found that the catalytic performance f PtNi@SWCNT nanoparticles are comparable.

**Table 3:** Comparison with the recent studies in the literature.

Catalyst	Conditions	Temp. (°C)	Time (h)	Yield^a^ (%)
γMnO_2_@GO^23^	BnOH (1.0 mmol), catalyst (10 mmol%), K_2_CO_3_ (0.5 mmol), toluene (3 ml)	80	3.0	91
CoL_2_@SMNP^14^	BnOH (1.0 mmol), catalyst (0.5 mol%), NHPI (1.0 mmol), CH_3_CN (3 ml), O_2_	70	16	97
Au–Cu/SiO_2_^42^	BnOH (0.36 mmol/min), catalyst (0.2 g), O_2_	260	2.0	98
Co-Bir^43^	BnOH (1.0 mmol), catalyst (50 mg), toluene (10 ml), O_2_	110	24	99
PdHAP-0^25^	BnOH (1.0 mmol), catalyst (0.1 g), trifluorotoluene (5 ml), O_2_	90	1.0	99
**PtNi@SWCNT nanoparticles (this study)**	BnOH (1.0 mmol), catalyst (0.04 mmol%), KOH (1.5 mmol), toluene (3 ml), O_2_	80	2.0	>99

^a^GC yield.

## Conclusions

In this paper, PtNi@SWCNT nanoparticles have been successfully synthesized using a simple single-step reduction method. PtNi@SWCNT nanoparticles are a promising material for utilization as a catalyst for alcohol oxidation, which is easy to synthesize, have a large surface area, and excellent catalyst properties. The results showed that the synthesized PtNi@SWCNT nanocomposites shown good catalytic performance for the conversion of BnOH species to the BA derivatives under mild conditions. The products were obtained within 2 hours in the presence of O_2_, by the very high selectivity (up to 100%). Herein, the catalytic reaction mechanism for the oxidation of BnOH with SWCNT was also proved. The results obtained in this study indicated an important role of these nanostructured carbon-supported bimetallic alloys in the oxidation of alcohols.

## Supplementary information

Supplementary Information.
