# Supplementary material for: Single-Walled Carbon Nanotube Supported PtNi Nanoparticles (PtNi@SWCNT) Catalyzed Oxidation of Benzyl Alcohols to the Benzaldehyde Derivatives in Oxygen Atmosphere
Source: Sci Rep. 2020 Jun 15;10:9656. doi: 10.1038/s41598-020-66492-x (PMC7295747; doi:10.1038/s41598-020-66492-x)
Supplement: Supplementary file 1 — Supplementary Information. [file 41598_2020_66492_MOESM1_ESM.docx]

**SUPPORTIVE INFORMATION**

**Single-Walled Carbon Nanotube Supported PtNi Nanoparticles (PtNi@SWCNT) Catalyzed Oxidation of Benzyl Alcohols to the Benzaldehyde Derivatives in Oxygen Atmosphere**

Haydar Göksu ^a*^, Kemal Cellat ^b^, Fatih Şen ^b*^

^a^Kaynasli Vocational College, Düzce University, Düzce 81900, Turkey

^b^Sen Research Group, Department of Biochemistry, Dumlupınar

University, 43100 Kütahya, Turkey

e-mail: haydargoksu@duzce.edu.tr (H. Göksu); fatih.sen@dpu.edu.tr (F. Şen)


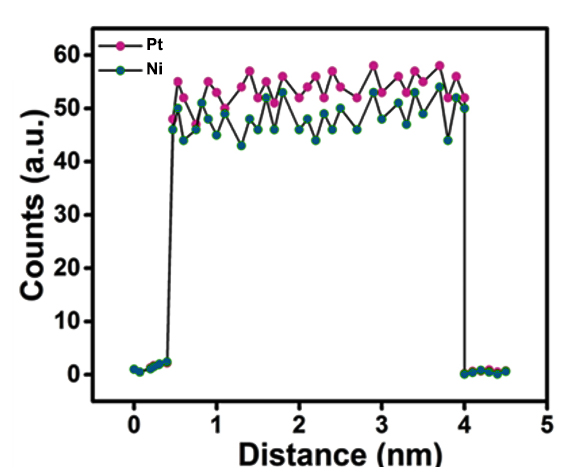


**Figure S1.** EELS line profile

**^1^H-NMR/^13^C-NMR Spectra for Oxidation Products**

**Benzaldehyde:** ^1^H NMR (400 MHz, CDCl_3_): *δ* 9.99 (s, 1H), 7.99 (dd, *J* = 5.9, 2.0 Hz, 2H), 7.64-7.56 (m, 1H), 7.54-7.45 (m, 2H). ^13^C NMR (100 MHz, CDCl_3_): *δ* 192.5, 136.4, 134.6, 129.8, 129.1.

**4-(dimethylamino)benzaldehyde:** ^1^H NMR (400 MHz, CDCl_3_): *δ* 9.76 (s, 1H), 7.78-7.70 (m, 2H), 6.74 (d, *J* = 8.9 Hz, 2H), 3.08 (s, 6H). ^13^C NMR (100 MHz, CDCl_3_): *δ* 190.5, 132.1, 111.5, 40.4.

**4-hydroxybenzaldehyde:** ^1^H NMR (400 MHz, CDCl_3_): *δ* 9.85 (s, 1H), 7.88-7.70 (m, 2H), 7.04-6.86 (m, 2H). ^13^C NMR (100 MHz, CDCl_3_): *δ* 191.3, 161.6, 132.6, 127.5, 116.1.

**3,4,5-trimethoxybenzaldehyde:** ^1^H NMR (400 MHz, CDCl_3_): *δ* 9.85 (s, 1H), 7.11 (s, 2H), 3.92 (s, 9 H). ^13^C NMR (100 MHz, CDCl_3_): *δ* 191.2, 153.7, 143.9, 131.8, 106.7, 61.1, 56.3.

**2,5-dimethoxybenzaldehyde:** ^1^H NMR (400 MHz, CDCl_3_): *δ* 10.43 (s, 1H), 7.31 (d, *J* = 3.3 Hz, 1H), 7.17-7.07 (m, 1H), 6.93 (d, *J* = 9.1 Hz, 1H), 3.88 (s, 3H), 3.78 (s, 3H). ^13^C NMR (100 MHz, CDCl_3_): *δ* 189.7, 156.8, 153.6, 123.6, 113.4, 110.4, 56.2, 55.9.

**
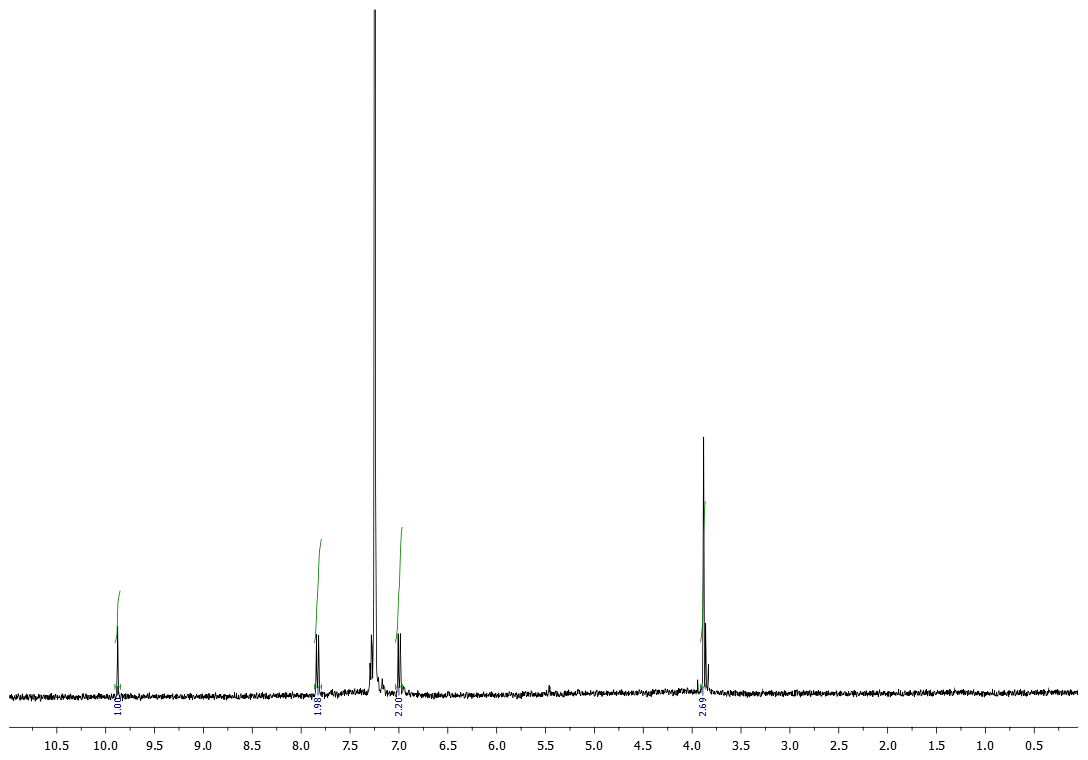
**

**4-methoxybenzaldehyde:** ^1^H NMR (400 MHz, CDCl_3_): *δ* 9.88 (s, 1H), 7.83 (d, *J* = 8.6 Hz, 2H), 7.00 (d, *J* = 8.6 Hz, 2H), 3.88 (s, 3H). ^13^C NMR (100 MHz, CDCl_3_): *δ* 190.5, 132.1, 111.5, 40.4.


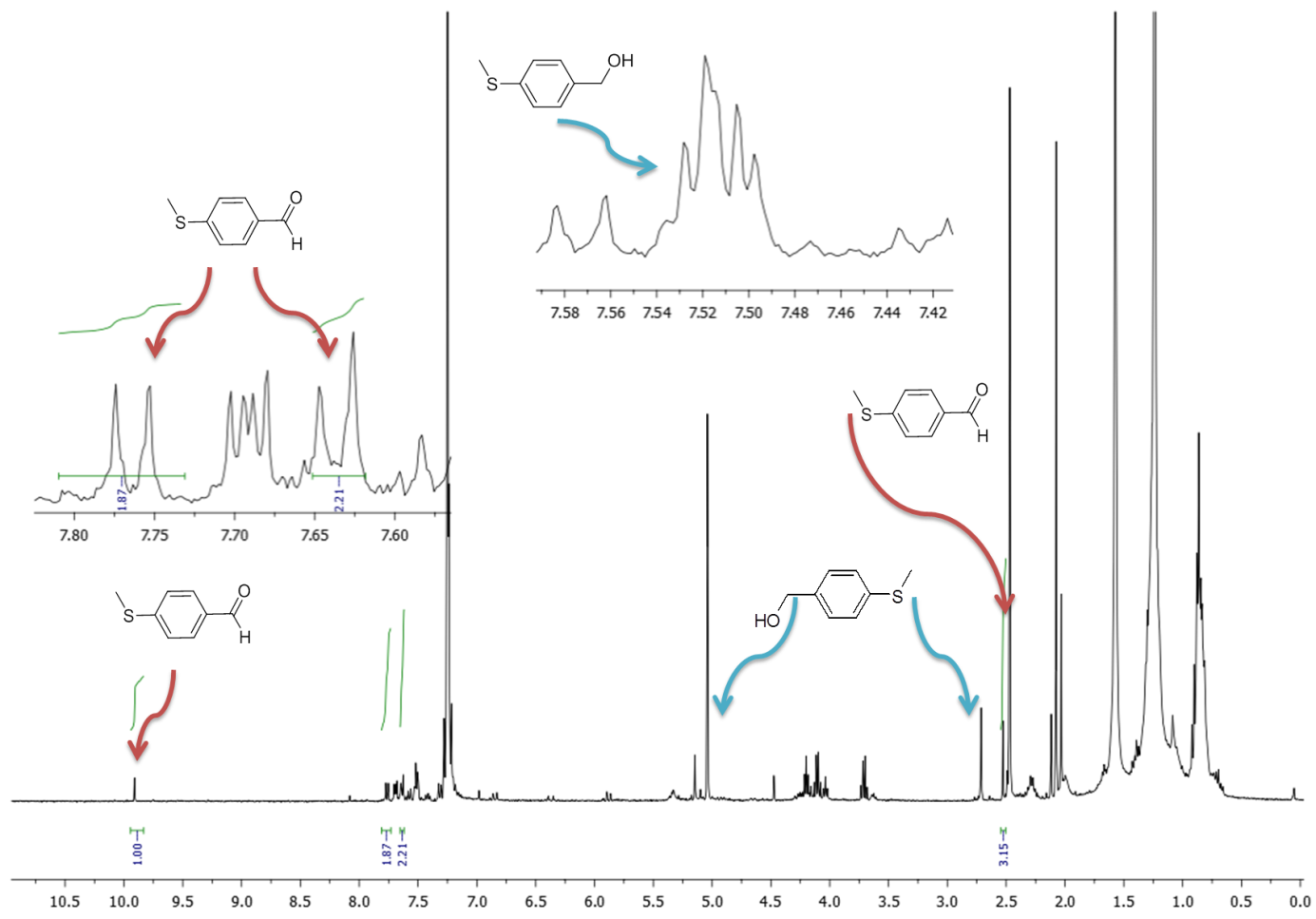


**4-(methylthio)benzaldehyde**

**4-methylbenzaldehyde:** ^1^H NMR (400 MHz, CDCl_3_): *δ* 9.95 (s, 1H), 7.76 (d, *J* = 8.0 Hz, 2H), 7.32 (d, *J* = 8.0 Hz, 2H), 2.42 (s, 3H). ^13^C NMR (100 MHz, CDCl_3_): *δ* 192.2, 145.7, 134.2, 129.9, 129.8, 21.9.

**4-(trifluoromethyl)benzaldehyde:** ^1^H NMR (400 MHz, CDCl_3_): *δ* 10.08 (s, 1H), 7.99 (d, *J* = 8.1 Hz, 2H), 7.79 (d, *J* = 8.1 Hz, 2H). ^13^C NMR (100 MHz, CDCl_3_): *δ* 191.2, 130.0, 126.2, 126.1.

**4-nitrobenzaldehyde:** ^1^H NMR (400 MHz, CDCl_3_): *δ* 10.15 (s, 1H), 8.51-8.29 (m, 2H), 8.20-7.91 (m, 2H). ^13^C NMR (100 MHz, CDCl_3_): *δ* 190.4, 140.1, 130.6, 124.4.

**2-fluorobenzaldehyde:** ^1^H NMR (400 MHz, CDCl_3_): *δ* 10.36 (s, 1H), 7.92-7.79 (m, 1H), 7.68-7.52 (m, 1H), 7.34-7.21 (m, 1H), 7.20-7.08 (m, 1H). ^13^C NMR (100 MHz, CDCl_3_): *δ* 187.4, 166.1, 136.5, 128.8, 124.7, 124.2, 116.7.

**4-fluorobenzaldehyde:** ^1^H NMR (400 MHz, CDCl_3_): *δ* 9.94 (s, 1H), 7.95-7.80 (m, 2H), 7.29-7.12 (m, 2H), 7.99 (d, *J* = 8.1 Hz, 2H), 7.79 (d, *J* = 8.1 Hz, 2H). ^13^C NMR (100 MHz, CDCl_3_): *δ* 190.6, 167.9, 165.3, 132.4, 132.3, 116.5, 116.3.

**4-bromobenzaldehyde:** ^1^H NMR (400 MHz, CDCl_3_): *δ* 9.96 (s, 1H), 7.77-7.71 (m, 2H), 7.70-7.64 (m, 2H). ^13^C NMR (100 MHz, CDCl_3_): *δ* 191.2, 131.9, 131.7, 131.1, 129.9.

**
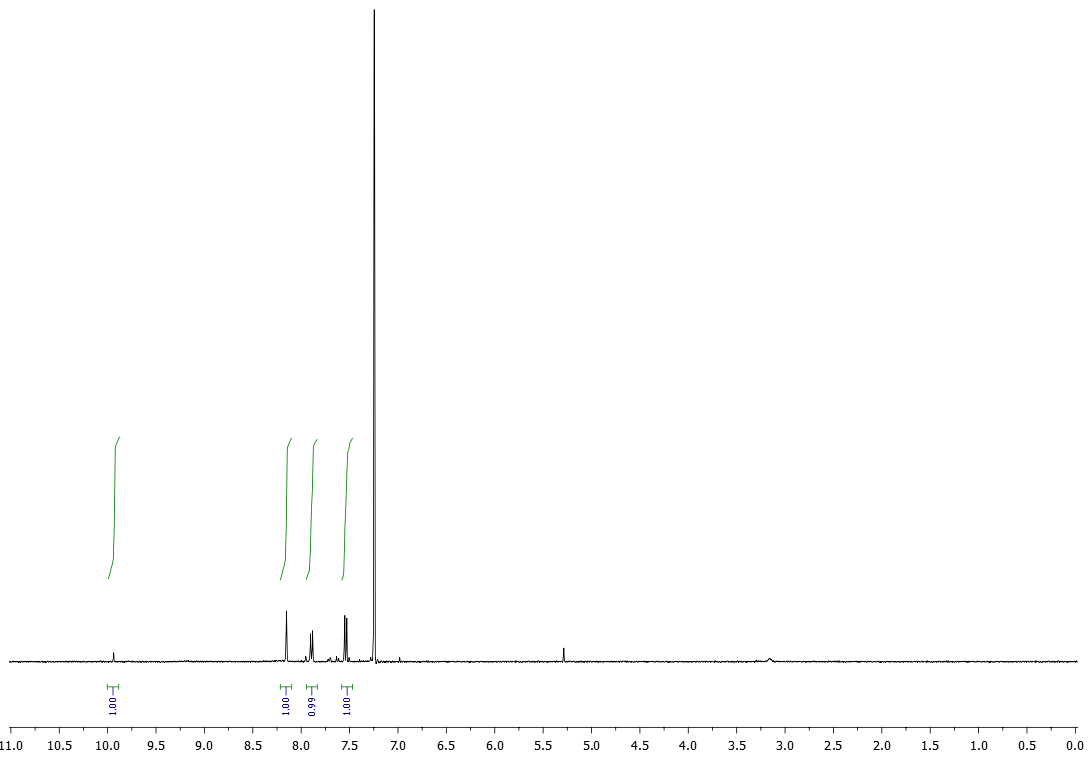
**


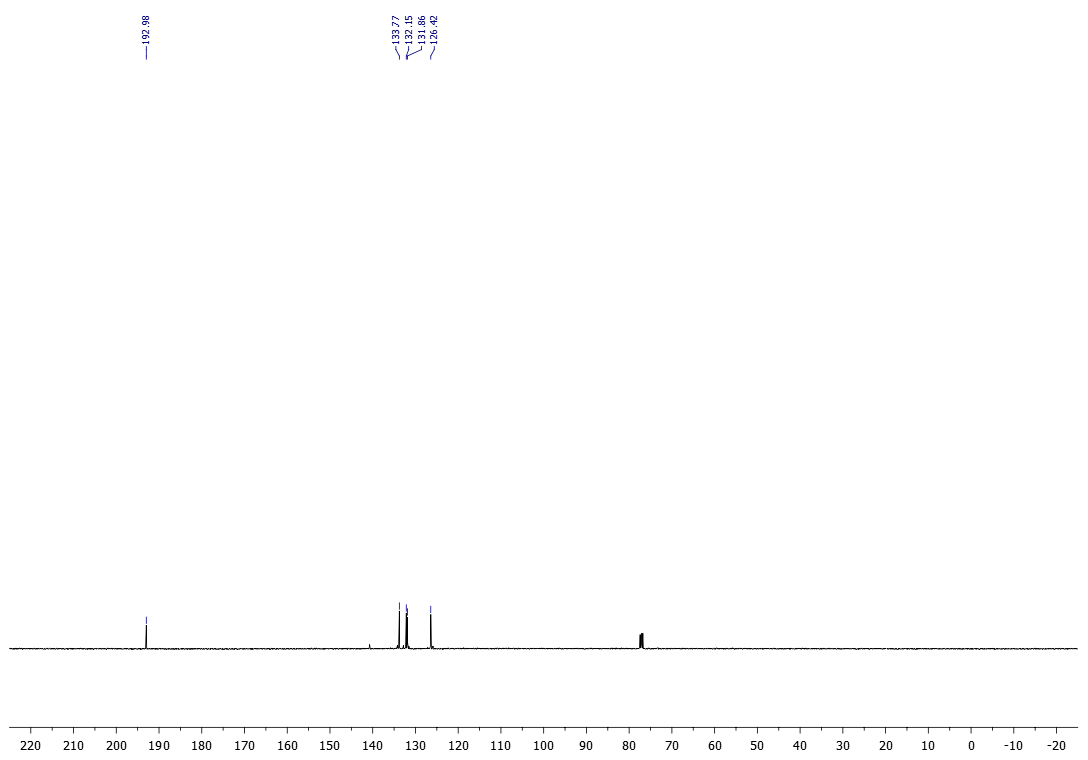


**3,4-dichlorobenzaldehyde:** ^1^H NMR (400 MHz, CDCl_3_): *δ* 9.94 (s, 1H), 8.15 (d, *J* = 2.0 Hz, 1H), 7.89 (dd, *J* = 8.4, 2.0 Hz, 1H), 7.53 (m, 1H). ^13^C NMR (100 MHz, CDCl_3_): *δ* 192.9, 133.8, 132.1, 131.9, 126.4.
